# Biallelic *RDH11* variants cause syndromic retinitis pigmentosa with early-onset cataracts and neurodevelopmental delay: a multicenter case series

**DOI:** 10.1038/s41431-026-02232-5

**Published:** 2026-09-16

**Authors:** Marianthi Karali, Susanne Kohl, Francesco Testa, Eleanor Hay, Yasas D. Kolambage, German Demidov, Tobias Haack, Hamid Ghaedi, Siying Lin, Gemma Natale, Vindya Subasinghe, Pyara Rathnayake, Sarah Wörz, Gavin Arno, Rebecca Buchert, Katarina Stingl, Francesca Simonelli, Sandro Banfi

**Affiliations:** 1 Department of Precision Medicine, Medical Genetics, University of Campania ‘Luigi Vanvitelli’, Naples, Italy; 2 Multidisciplinary Department of Medical, Surgical and Dental Sciences, Eye Clinic, University of Campania ‘Luigi Vanvitelli’, Naples, Italy; 3 Telethon Institute of Genetics and Medicine, Pozzuoli, Italy; 4 Institute for Ophthalmic Research, Centre for Ophthalmology, Tübingen, Germany; 5 Department of Clinical Genetics, Great Ormond Street Hospital for Children, London, UK; 6 Department of Anatomy, Faculty of Medicine, Sabaragamuwa University of Sri Lanka, Ratnapura, Sri Lanka; 7 Institute of Medical Genetics and Applied Genomics, University of Tübingen, Tübingen, Germany; 8 Centre for Rare Diseases, University of Tübingen, Tübingen, Germany; 9 Department of Pathology and Molecular Medicine, Queen’s University, Kingston, ON Canada; 10 Manchester Centre for Genomic Medicine and Department of Ophthalmology, Manchester University NHS Foundation Trust, Manchester, UK; 11 Division of Evolution, Infection and Genomics, School of Biological Sciences, Faculty of Biology, Medicine and Health, University of Manchester, Manchester, UK; 12 National Institute of Health Research Biomedical Research Centre at Moorfields Eye Hospital, The UCL Institute of Ophthalmology, London, UK; 13 Lady Ridgeway Hospital for Children, Colombo, Sri Lanka; 14 UCL Institute of Ophthalmology, University College London, London, UK; 15 Division of Research, Greenwood Genetic Center, Greenwood, SC USA; 16 University Eye Hospital, Centre for Ophthalmology, University of Tübingen, Tübingen, Germany

**Keywords:** Disease genetics, Genetics research, Retina

## Abstract

Biallelic variants in the *RDH11* gene, a retinol dehydrogenase involved in the visual cycle and systemic retinoid homeostasis, were initially implicated in a rare condition characterized by retinal dystrophy, early-onset cataract, neurodevelopmental anomalies and myopathy, through single-family reports, with this association more recently being confirmed in a larger study. Here, we further establish the pathogenic role of *RDH11* by presenting a large multi-center cohort, comprising eight individuals from seven unrelated families. Comprehensive genetic analysis identified homozygous variants in all affected subjects, including two novel variants, strongly supporting loss-of-function as the primary disease mechanism. Detailed clinical phenotyping defined a consistent and severe multisystem disorder. Ophthalmologically, the hallmark features include bilateral congenital or early-childhood cataracts requiring surgical intervention, accompanied by retinitis pigmentosa (RP). In terms of extra-ocular involvement, the cohort exhibited a high prevalence of neurodevelopmental delay, including intellectual disability, autistic spectrum disorder and learning difficulties. These features were frequently accompanied by congenital microcephaly, intrauterine and postnatal growth restriction, facial dysmorphisms, and dental anomalies. By significantly expanding both the mutational and phenotypic spectrum of *RDH11*-related disease, our findings provide independent replication of this gene-disease association and definitively support *RDH11* as a bona fide syndromic RP gene.

## Introduction

Short‑chain dehydrogenase/reductase (SDR) enzymes in the retina and retinal pigment epithelium (RPE) coordinate the cycling between retinol and retinaldehyde forms of vitamin A derivatives (retinoids) [1]. Disruption of these enzymes can cause inherited retinal diseases (IRDs) by perturbing chromophore availability and retinoid balance [1, 2].

Retinol dehydrogenase 11 (*RDH11*), a member of the SDR family, is a 318-amino-acid microsomal enzyme that uses NADPH/NADP⁺ and acts on several retinoid isomers (all-trans, 9-cis, 11-cis), showing a preference for converting retinal into retinol rather than the reverse [1, 3]. *RDH11* was initially identified as *PSDR1*, an androgen‑regulated transcript highly expressed in prostate epithelium, with RNA in situ hybridisation confirming high expression in the prostate and lower-level expression across other tissues, including liver, kidney, retina, and testis [4] (http://www.proteinatlas.org/ENSG00000072042-RDH11/tissue), consistent with a housekeeping role in retinoid metabolism beyond the eye [5]. RDH11 shares conserved SDR motifs for coenzyme binding and catalysis [3]. Biochemically, human RDH11 displays strong activity toward all‑trans‑, 9‑cis‑, and 11‑cis‑retinol, minimal activity on toxic lipid peroxidation products, and no detectable dehydrogenase activity toward steroids – features that differentiate it from some SDR paralogs [3]. In the eye, RDH11 and the closely linked paralog RDH12 (encoded in antisense orientation ∼30 kb apart on 14q24.1) form part of a subfamily of dual‑substrate retinol dehydrogenases implicated in the visual cycle across photoreceptors and RPE [3].

Preclinical data support a conserved role of RDH11 in retinoid handling outside the retina. In mice, *Rdh11* is expressed in liver and testis; loss‑of‑function reduces the conversion of all‑trans‑retinaldehyde to all‑trans‑retinol and lowers tissue retinol levels under vitamin‑A-limited conditions, indicating a physiological role in maintaining retinoid pools [5]. In the retina, the relatively constant expression of *RDH11* compared with the developmentally regulated expression of *RDH12* suggests RDH11 performs a basal, housekeeping function that may buffer retinoid flux under stress [6]. Together, these mechanistic and animal model data strengthen the biological plausibility for human phenotypes arising from biallelic *RDH11* variants.

Despite a well‑defined molecular function, the human disease association of *RDH11* has remained uncertain (OMIM #616108) until recently. The first clinical report described an Italian‑American sibship with biallelic nonsense variants (p.Arg67Ter, p.Arg108Ter), presenting with rod‑cone dystrophy, early cataracts, short stature, neurodevelopmental features, and characteristic fundus changes with predominant loss of scotopic electroretinogram (ERG) response [7]. A similar syndromic retinitis pigmentosa (RP) with cataracts and mild myopathy was later reported in another biallelic *RDH11* patient [8]. A third subject with autism, microcephaly, dysmorphisms and oligodontia was subsequently described, but this patient had normal ERG responses and no visual complaints, despite the presence of yellow deposits at the RPE level [9]. A recent study on nine families reported that biallelic loss-of-function variants in *RDH11* cause a distinct syndrome characterised primarily by juvenile-onset progressive muscle weakness, often preceded by asymptomatic hyperCKemia, vision loss, and neurodevelopmental delays [10].

The association between biallelic *RDH11* variants and syndromic features (OMIM #616108), previously limited to a candidate classification due to sparse evidence, is therefore becoming more established as the number of characterised cohorts gradually increases. Our study definitively confirms the pathogenic role of *RDH11* by reporting seven unrelated families with biallelic variants in this gene, integrating comprehensive ophthalmological assessments and clinical evaluations.

## Subjects and methods

Clinical, ophthalmological and genetic data were collected retrospectively across participating centres, and full methodological details, including recruitment criteria, imaging protocols and sequencing pipelines, are provided in Supplementary File 1.

## Results

### Case series overview

The cohort comprised eight affected subjects (5 males, 3 females; age range: 9–63 years) from seven unrelated families of Italian (*n* = 3 from 2 families), German (*n* = 1), Turkish (*n* = 1), British (*n* = 1), Sri Lankan (*n* = 1) and Iranian (*n* = 1) origins. All patients carried homozygous pathogenic variants in *RDH11*. Demographic and clinical features are summarised in Table 1. An overview of the *RDH11* causative variants identified in this case series or already reported in the literature is shown in Table 2.

**Table 1:** Demographic, genetic and clinical features of subjects with *RDH11*-related disorder.

Family	Patient	Sex	Age	Familial/sporadic	Nucleotide change	Protein change	Zygosity	Developmental abnormalities	Other symptoms
F1	P1	M	44	familial	c.199C>T	p.Arg67Ter	Hom.	developmental delay, language skills delay, cognitive delay	icterus at birth (IV grade), microcephaly, micrognathia, protruding ears (free ear lobe), syncope at 19 y, asymptomatic hyperCKemia
F1	P2	F	38	familial	c.199C>T	p.Arg67Ter	Hom.	developmental delay, cognitive delay	microcephaly, micrognathia, protruding ears (free ear lobe)
F2	P3	F	31	sporadic	c.235G>T	p.Glu79Ter	Hom.	not reported	microcephaly, midface hypoplasia, dorsal hyperkyphosis, bilateral hallux valgus, ovarian endometriosis
F3	P4	M	62	sporadic	c.749G>A	p.Trp250Ter	Hom.	developmental delay, psychomotoric development delay	microcephaly, spasms since infancy, lung emboly at 57 y
F4	P5	M	37	sporadic	c.688_689insGC	p.Val230GlyfsTer18	Hom.	developmental delay	preterm born, thromboses
F5	P6	M	9	sporadic	c.455G>A	p.Gly152Asp	Hom.	global developmental delay, autistic spectrum disorder, ADHD, intellectual disability, poor coordination	sickle cell anaemia (HbSC), IUGR (Birth wt: -3SD), significant growth restriction (aged 6, ht: -3SD, wt: -5.5SD), severe microcephaly (head circumference: -6SD), small right frontal subcortical nodular grey matter heterotopia, thin corpus callosum, mild volume loss cerebellum, afebrile seizures, sparse and coarse hair, retractile testes
F6	P7	M	11	sporadic	c.601_608del	p.Ala201SerfsTer35	Hom.	global developmental delay, moderate intellectual disability	severe microcephaly, short stature, failure to thrive, craniosynostosis, sloping forehead, abnormality of the maxilla, abnormal facial shape, abnormal dentition, prominent ears, sandal gap
F7	P8	F	n/a	familial	c.413C>T	p.Thr138Ile	Hom.	intellectual disability	seizures

*ADHD* attention-deficit/hyperactivity disorder, *ht* height, *IUGR* intrauterine growth restriction, *n/a* not available, *SD* standard deviation, *wt* weight.

**Table 2:** Ophthalmological findings in subjects with *RDH11*-related disorder.

Family	Patient	Sex	Ophthalmological abnormalities	Reported onset of visual symptoms (latest visit)	BCVA	Retinal findings	SD-OCT	ERG
F1	P1	M	RP (typical), bilateral cataracts	22 (44)	20/33 RE 20/30 LE	Diffuse RPE dystrophy with rare pigment accumulations in mid-periphery. Yellow RPE deposits in mid-periphery. Diffuse macular hypoAF and hyperAF with atrophic spots.	RPE dystrophy with some intraretinal microcysts. Patchy residual EZ.	Scotopic responses non-recordable. Photopic responses subnormal.
F1	P2	F	RP (typical), CME (treated), bilateral cataracts	10 (38)	20/30 RE 20/25 LE	Diffuse RPE dystrophy with rare pigment accumulations in mid-periphery. Irregular areas of increased AF mixed with small hypoAF foci at the parafoveal region. Foveal sparing.	Progressive ONL thinning and EZ disruption outside the fovea.	Scotopic responses non-recordable. Photopic responses subnormal.
F2	P3	F	RP (atypical), CME, bilateral cataracts	5 (31)	20/80 RE 20/100 LE	Diffuse RPE dystrophy with areas of depigmentation and hyperpigmentation along the retinal vasculature in mid-periphery.	HyperAF ring around fovea. Almost complete loss of EZ band. CME. Epiretinal membrane.	Scotopic responses non-recordable. Photopic responses subnormal.
F3	P4	M	RP, bilateral cataracts	6-7	Blind RE 20/80 LE	Retinal dystrophy. Retinal detachment in LE.	Epiretinal membrane and cystic retinoschisis in the macula.	Dark-adaptation thresholds pathologically elevated and mixed (cone/rod mediated).
F4	P5	M	RP, bilateral cataracts	5	20/40 RE 20/32 LE	Retinal dystrophy	Small remnants of foveal photoreceptor layering.	Scotopic responses non-recordable. Photopic responses non-recordable.
F5	P6	M	RP, bilateral zonular developmental cataracts	6	(6 y): 20/160 RE; 20/320 LE (9 y): 20/50 RE; 20/50 LE	Retinal dystrophy mild (dark/grey retina).	Outer retinal degeneration with loss of photoreceptor layer/EZ outside the fovea and a small central area of preserved subfoveal outer retina. No CME.	Generalised retinal dysfunction (rods>cones). In VEP BL: macular pathway dysfunction with some macular preservation.
F6	P7	M	RP, cataracts, exotropia, gaze-evoked nystagmus	7	20/80 RE 20/80 LE	Retinal dystrophy	Mild thinning of the outer retinal layers. Subtle EZ band irregularities. Preserved foveal architecture.	n/a
F7	P8	F	cataracts	n/a	n/a	n/a	n/a	n/a

Ages are reported in years.

*AF* autofluorescence, *BCVA* best corrected visual acuity, *CME* cystoid macular edema, *ERG* electroretinogram, *EZ* ellipsoid zone, *LE* left eye, *n/a* not available, *ONL* outer nuclear layer, *RE* right eye, *RP* retinitis pigmentosa, *SD-OCT* spectral-domain optical coherence tomography, *VEP BL* visual evoked potential bilateral.

### Clinical presentation and molecular characterisation of subjects with *RDH11-*related disease

#### Family 1: patients P1 and P2

##### Clinical findings in P1

**Fig. 1:**
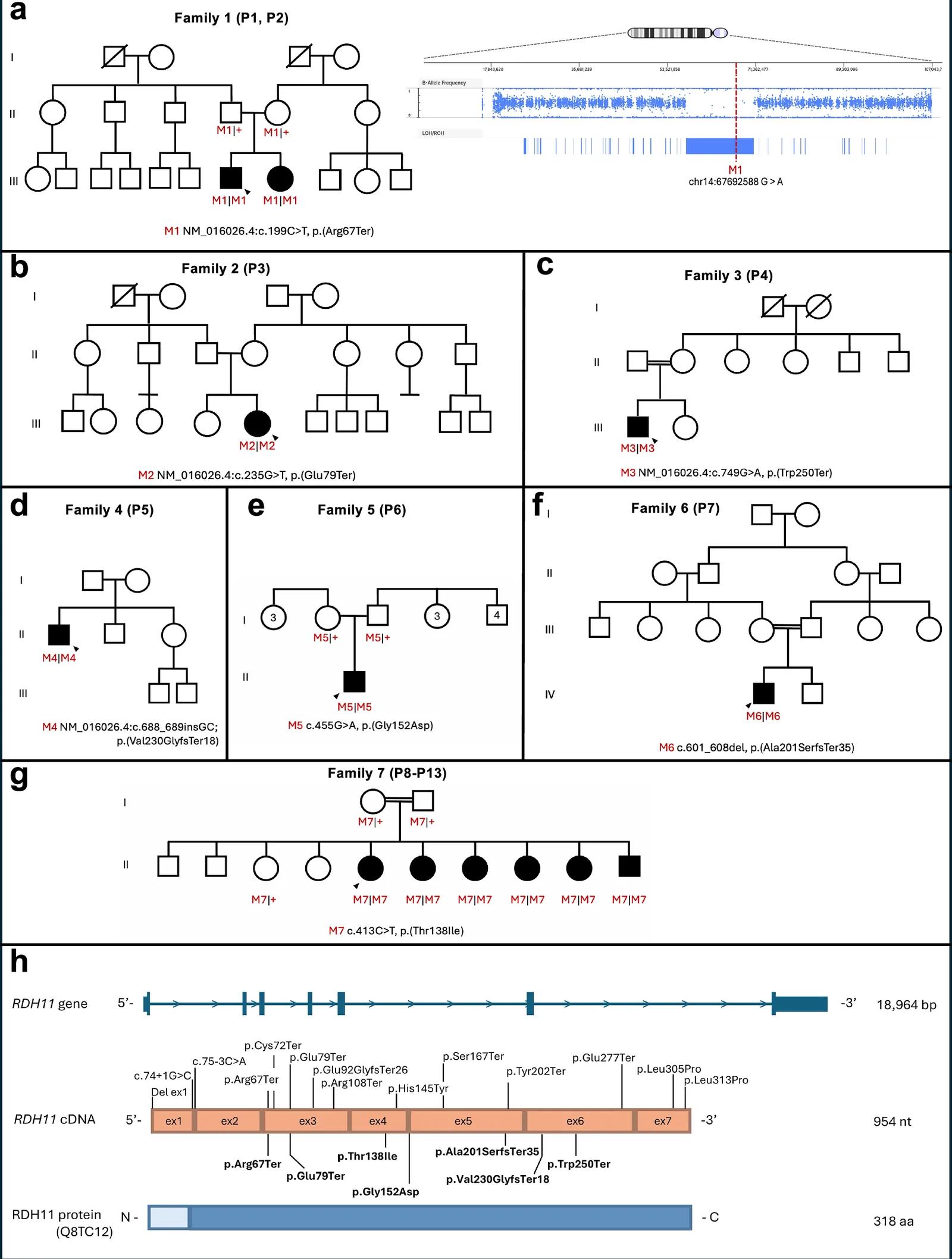
Pedigrees and variants identified in the *RDH11* cohort. **a**–**g** Pedigrees of the families included in this study and segregation analysis of the identified disease-causing variants (performed whenever parental samples were available). Probands are indicated by arrowheads. Filled symbols denote affected individuals with syndromic RP. Variant positions are reported to the RefSeq transcript sequence NM_016026.4 (*RDH11*). Homozygosity mapping of P1 showing the proportion of sequencing reads supporting an alternate allele (dots in ‘B-Allele frequency’) and regions of homozygosity (blue bars in ‘LOH/ROH’) across chromosome 14 and the genomic location of M1 (red dotted line) is shown in **a** (right). **h** Schematic representation of the relative position of the variants identified in this study across the *RDH11* cDNA (variants depicted in bold). Variants reported in literature [7–10] are depicted in normal font (shown above). The *RDH11* gene structure and protein domains are also shown.

Patient P1, a Caucasian male, was referred to the eye clinic at the age of 32 years (y) with complaints of night blindness and decreased visual acuity since the age of 28. He is the first of two affected siblings born to reportedly non-consanguineous parents (Fig. 1a). The proband was born at term following an uncomplicated pregnancy and presented with grade IV jaundice at birth. Physical examination had revealed congenital microcephaly, but no other dysmorphic features (Table 1). Speech and language milestones were delayed, and a neurological assessment indicated a severe cognitive delay (intelligence quotient (IQ) of 70 with good memory skills). At his most recent physical examination (44 y), he presented microcephaly, micrognathia and protruding ears with free earlobes. His hearing was normal and no teething problems were reported (besides orthodontic treatment to correct dental issues attributed to prolonged pacifier use).

His systemic history began at the age of 16 with asymptomatic elevated creatine phosphokinase (CK) levels, leading to an inconclusive neurological workup. At the age of 19, he experienced syncope, and investigations revealed sinus node dysfunction with bradycardic pauses, leading to the implantation of a permanent pacemaker. Extensive investigations, including multiple muscle biopsies and genetic tests for mitochondrial disorders, were negative.

**Fig. 2:**
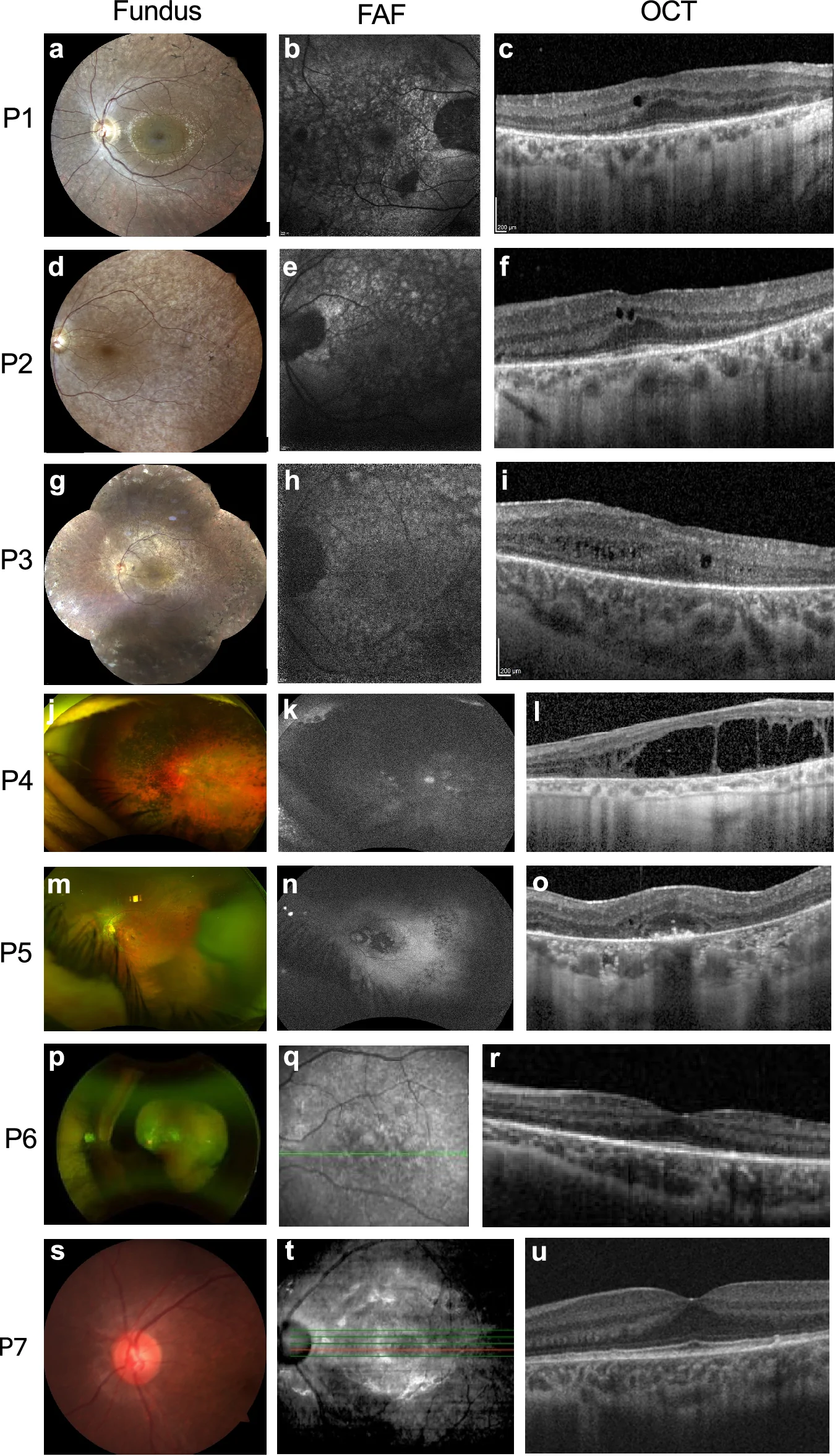
Multimodal retinal imaging in patients with biallelic *RDH11* variants. Colour fundus photography (left side of each panel), fundus autofluorescence imaging (FAF, central image of each panel), and macular spectral-domain optical coherence tomography (SD-OCT, right side of each panel) of the subjects included in this study. **a**–**c** *Patient P1:* Fundus photograph showing diffuse retinal pigment epithelium (RPE) dystrophy with mixed typical and atypical pigment in mid-periphery. FAF shows foveal sparing while the parafoveal region presents irregular areas of increased AF foci mixed with small hypoAF foci. SD-OCT reveals macular thinning with patchy residual ellipsoid-zone (EZ) integrity and small intraretinal cystoid spaces. **d**–**f** *Patient P2:* Colour fundus image shows diffuse RPE dystrophy with typical and atypical pigment deposits in mid-periphery. FAF reveals relative foveal sparing with an irregular parafoveal pattern of increased autofluorescence mixed with small hypoautofluorescent foci. SD-OCT reveals a moderate macular thinning with partial preservation of the EZ at the foveal area, progressive thinning of the ONL, EZ disruption outside the fovea and small intraretinal cystoid spaces. **g**–**i** *Patient P3:* Colour fundus image showing diffuse RPE dystrophy with areas of depigmentation predominantly along the retinal vasculature and with mixed typical and atypical pigment in periphery. FAF demonstrates widespread hypoautofluorescence, with relative foveal sparing, while SD-OCT reveals diffuse outer retinal thinning, near-complete disruption of the EZ band, cystoid macular edema, and an epiretinal membrane. **j**–**l** *Patient P4:* Colour fundus image shows dense mid peripheral pigmentary retinal atrophy with profoundly attenuated retinal vessels. FAF shows a very reduced signal, partially due to profound retinal atrophy and partly due to the hyperautofluorescent signal of the optic nerve head drusen. In the SD-OCT of the left eye, central retinal schisis is evident. **m**–**o** *Patient P5:* Colour fundus image shows slight pigmentary changes in mid-periphery and in the centre, along with attenuated retinal vessels. In the FAF image the pigmentary changes correspond to central and mid-peripheral atrophic areas. The SD-OCT on the central macula reveals atrophy of the outer retina with a small foveal area of partially preserved, albeit degenerating, photoreceptors. **p**–**r** *Patient P6:* Ultrawide-field pseudocolour imaging (**p**) did not show any overt fundus abnormality, aside from a subtly grey appearance of the retina. SD-OCT infrared reflectance imaging (**q**) showed mottled hypo- and hyper-reflective change. The corresponding horizontal B-scan (**r**) demonstrated outer retinal degeneration with loss of the photoreceptor layer/EZ outside the fovea, with a small central area of preserved subfoveal outer retina. No cystoid macular edema was present. **s**–**u** *Patient P7:* Colour fundus image (**s**) reveals a normal optic disc and a relatively preserved posterior pole, with subtle RPE mottling and mild vascular attenuation. AF imaging (**t**) shows a patchy and heterogeneous autofluorescence pattern at the posterior pole, with irregular areas of increased and decreased autofluorescence, consistent with early RPE dysfunction. SD-OCT (**u**) shows preserved foveal architecture, with mild thinning of the outer retinal layers and subtle irregularity of the EZ band.

His visual symptoms began at the age of 22. At his most recent evaluation (44 y), his best-corrected visual acuity (BCVA) was 20/33 in the right eye (RE) and 20/30 in the left (LE) (Table 2). Slit-lamp examination revealed bilateral pseudophakia. Fundus examination revealed diffuse RPE dystrophy with both typical and atypical pigment accumulation in mid-periphery, and numerous yellow RPE deposits visible mostly in mid-peripheral retina (Fig. 2a, and Supplementary Fig. 1). Fundus autofluorescence (FAF) imaging showed foveal sparing, while the parafoveal region presented irregular areas of increased autofluorescence with small hypoautofluorescent foci (Fig. 2b). Spectral-domain optical coherence tomography (SD-OCT) of the macula revealed macular thinning with a patchy residual ellipsoid zone (EZ) and small intraretinal cystoid spaces (Fig. 2c). Goldmann visual field (GVF) testing showed constricted isopters. Microperimetry documented stable fixation with a reduced mean sensitivity (14.9 dB RE, 10.2 dB LE). Full-field electroretinogram (ffERG) demonstrated severe rod-cone dysfunction, with non-detectable scotopic and subnormal photopic responses (Table 2). These findings led to a diagnosis of RP, according to established diagnostic criteria [11]. In the annual follow-up visits over the last 10 years, his BCVA remained relatively stable, without significant decline over the observation period. In contrast, his microperimetry values gradually declined. The most significant change was observed in the photopic ffERG response, which remained stable until around the age of 37, after which it sharply declined (average reduction rate: −2.8 µV/year).

##### Clinical findings in P2

The proband’s younger sister, P2, presented similar ophthalmological and neurodevelopmental features (Fig. 1a). She was diagnosed with cognitive delay (IQ of 70, memory problems, poor school performance), less pronounced compared to that of her brother. She presented with congenital microcephaly, micrognathia and protruding ears with free earlobes (Table 1). Teething, hearing and gait were reported as normal, and she did not report other systemic involvements like her brother.

She was diagnosed with congenital cataracts at the age of 10 y (Table 2). At 22 y, ffERG indicated significant retinal dysfunction. She underwent bilateral cataract surgery at the age of 27 and was diagnosed with cystoid macular edema (CME) (28 y), successfully treated with acetazolamide. At the most recent evaluation (38 y), her BCVA was 20/30 (RE) and 20/25 (LE), with bilateral pseudophakia. Multimodal retinal imaging revealed diffuse RPE dystrophy (detailed in Fig. 2d–f). GVF testing showed constriction to the central 20°. Microperimetry documented stable fixation (mean sensitivity: 21.0 dB RE, 22.2 dB LE), while ffERG demonstrated severe rod-cone dysfunction, with non-recordable scotopic and subnormal photopic responses, a profile nearly identical to that of her brother.

Longitudinal data from annual follow-ups over a 10-year period revealed a clear disease progression. BCVA remained well preserved (0.9) for the first 7 years of follow-up before declining to 0.6, where it stabilised. Microperimetry showed a fluctuating, but overall declining trend. The progression of the ffERG was more severe (average rate: −1.9 µV/year) during the entire follow-up period, with a reduction in the amplitude of the photopic response starting in her mid-thirties.

##### Molecular findings for family 1

Genome Sequencing (GS) of P1 identified a homozygous nonsense variant (NM_016026.4:c.199C>T; p.Arg67Ter) in the *RDH11* gene (M1, Fig. 1a,h), predicted to introduce a premature termination codon (PTC) at amino acid 67. Mutant transcripts are predicted to undergo nonsense-mediated decay (NMD). No other variants in IRD-associated genes were sufficient to account for the observed phenotype. Sanger sequencing confirmed this variant in the homozygous state in the affected sister (P2), while both parents were heterozygous carriers. The variant lies within a large region of homozygosity (ROH) on chromosome 14 (Fig. 1a, *right*), suggesting distant consanguinity. It is classified as pathogenic according to the ACMG criteria (PVS1_v.strong/PM2_supporting/PM3_supporting/PP1_supporting/PP3_supporting/PP4_supporting), has an allele frequency (AF) of 9×10^-6^ in gnomAD and was previously reported in compound heterozygosity with another nonsense variant in the first family reported with *RDH11* disease [7] (Table 3).

**Table 3:** Overview of *RDH11* variants identified in our cohort and reported in literature as pathogenic/likely pathogenic.

cDNA change^†^	Protein change	Functional class	ACMG classification of the *RDH11* variants reported in this study ^£^	ACMG criteria ^$^	gnomAD frequency (v4.1.1)	CADD	REVEL	Mutscore	dbscSNV*	SpliceAI^€^	Reference	ClinVar ^‡^
c.74+1G>C	p.?	Splicing	⁃	⁃	0.000002053	35	n/a	n/a	ada: 0.99996; rf: 0.95	AL: 0.01 DL: 0.99 AG: 0.00 DG: 0.53	[10]	not listed
c.75-3C>A	p.?	Splicing	⁃	⁃	not present	14.93	n/a	n/a	ada:0.80237; rf: 0.468	AL: 0.50 DL: 0.34 AG: 0.67 DG: 0.03	[8]	not listed
exon 1 del	?	Deletion	⁃	⁃	not present	n/a	n/a	n/a	n/a	n/a	[10]	not listed
c.199C>T	p.Arg67Ter	Nonsense	Pathogenic	PVS1_VERY STRONG PM2_SUPP PM3_SUPP PP1_SUPP PP3_SUPP PP4_SUPP	0.000009163	38	n/a	n/a	n/a	AL: 0.06 DL: 0.07 AG: 0.04 DG: 0.00	this study (M1) & [7, 10]	VCV000161115.10 (P)
c.216C>A	p.Cys72Ter	Nonsense	⁃	⁃	not present	35	n/a	n/a	n/a	AL: 0.02 DL: 0.01 AG: 0.02 DG: 0.00	[9]	not listed
c.235G>T	p.Glu79Ter	Nonsense	Pathogenic	PVS1_VERY STRONG PM2_SUPP PM3_SUPP PP3_SUPP PP4_SUPP	0.000000687	36	n/a	n/a	n/a	AL: 0.02 DL: 0.01 AG: 0.01 DG: 0.00	this study (M2) & [10]	not listed
c.269_273dup	p.Glu92GlyfsTer26	Frameshift	⁃	⁃	not present	n/a	n/a	n/a	n/a	AL: 0.06 DL: 0.02 AG: 0.00 DG: 0.00	[10]	not listed
c.284T>A	p.Leu95Ter	Nonsense	⁃	⁃	not present	38	n/a	n/a	n/a	AL: 0.14 DL: 0.09 AG: 0.00 DG: 0.16	none	VCV003248968.1 (P)
c.322C>T	p.Arg108Ter	Nonsense	⁃	⁃	0.00001300	39	n/a	n/a	n/a	AL: 0.17 DL: 0.07 AG: 0.00 DG: 0.04	[7]	VCV000161116.10 (P)
c.413C>T	p.Thr138Ile	Missense	Likely Pathogenic	PM2_SUPP PM3_SUPP PP1_SUPP PP3_SUPP PP4_SUPP	0.00001642	31	Path. Supporting (0.704)	0.804	n/a	AL: 0.01 DL: 0.00 AG: 0.00 DG: 0.00	this study (M7)	VCV001919223.2 (VUS)
c.433C>T	p.His145Tyr	Missense	⁃	⁃	0.000000685	28.5	Uncertain (0.57)	0.585	n/a	AL: 0.01 DL: 0.00 AG: 0.00 DG: 0.00	[10]	not listed
c.455G>A	p.Gly152Asp	Missense/Splicing	Likely Pathogenic	PM2_SUPP PM3_SUPP PP3_VERY STRONG PP4_SUPP	0.00000479	34	Path. Moderate (0.918)	0.89	ada: 0.99926; rf: 0.924	AL: 0.03 DL: 0.03 AG: 0.04 DG: 0.00	this study (M5)	VCV001475158.6 (VUS)
c.484G>T	p.Glu162Ter	Nonsense	⁃	⁃	not present	37	n/a	n/a	n/a	AL: 0.02 DL: 0.01 AG: 0.04 DG: 0.00	none	VCV000842721.7 (P)
c.500C>G	p.Ser167Ter	Nonsense	⁃	⁃	0.000000684	37	n/a	n/a	n/a	AL: 0.01 DL: 0.01 AG: 0.01 DG: 0.00	[10]	not listed
c.601_608del	p.Ala201SerfsTer35	Frameshift	Pathogenic	PVS1_VERY STRONG PM2_SUPP PM3_SUPP PP4_SUPP	not present	n/a	n/a	n/a	n/a	AL: 0.11 DL: 0.07 AG: 0.01 DG: 0.02	this study (M6)	not listed
c.606C>A	p.Tyr202Ter	Nonsense	⁃	⁃	not present	33	n/a	n/a	n/a	AL: 0.11 DL: 0.15 AG: 0.00 DG: 0.03	[10]	not listed
c.688_689insGC	p.Val230GlyfsTer18	Frameshift	Pathogenic	PVS1_VERY STRONG PM2_SUPP PM3_SUPP PP4_SUPP	not present	n/a	n/a	n/a	n/a	AL: 0.00 DL: 0.00 AG: 0.06 DG: 0.04	this study (M4)	not listed
c.749G>A	p.Trp250Ter	Nonsense	Pathogenic	PVS1_VERY STRONG PM2_SUPP PM3_SUPP PP3_SUPP PP4_SUPP	0.00000752	35	n/a	n/a	n/a	AL: 0.24 DL: 0.29 AG: 0.00 DG: 0.00	this study (M3)	VCV002446452.1 (LP)
c.829G>T	p.Glu277Ter	Nonsense	⁃	⁃	not present	38	n/a	n/a	n/a	AL: 0.35 DL: 0.36 AG: 0.00 DG: 0.00	[10]	not listed
c.914T>C	p.Leu305Pro	Missense	⁃	⁃	not present	29.4	Path. Moderate (0.83)	0.935	n/a	AL: 0.00 DL: 0.00 AG: 0.05 DG: 0.00	[10]	not listed
c.938T>C	p.Leu313Pro	Missense	⁃	⁃	not present	28.7	Path. Moderate (0.885)	0.829	n/a	AL: 0.00 DL: 0.00 AG: 0.04 DG: 0.00	[8]	not listed

*n/a* not available.

† Position refers to the RefSeq NM_016026.4.

£ ACMG classification (manual).

$ Manually assigned ACMG criteria.

* dbscSNV scores for variants near canonical or cryptic splice sites (ada, AdaBoost; rf, Random Forest).

€ SpliceAI score (http://spliceailookup.broadinstitute.org; AL, acceptor loss; DL, donor loss; AG, acceptor gain; DG, donor gain).

‡ SNV Variants reported in ClinVar as Pathogenic or Likely Pathogenic, and entries related to variants reported in our cohort (regardless of their ClinVar classification).

#### Family 2: patient P3

##### Clinical findings in P3

P3, a 31-year-old Caucasian female, is a sporadic patient, with unremarkable family history, born to reportedly non-consanguineous parents (Fig. 1b). She was delivered at term by cesarean section due to intrauterine growth retardation and fetal microcephaly (birth weight 2500 g; no perinatal or neonatal problems) (Table 1). At her latest physical examination, she was 151 cm tall, weighed 50 kg, and had a head circumference of 50 cm (microcephaly). She also presented midface hypoplasia, dorsal hyperkyphosis, bilateral hallux valgus, and ovarian endometriosis. The oral cavity and abdominal organs were within normal limits.

Strabismus and frequent falls were noted in childhood, leading to a diagnosis of severe astigmatism. By the age of 13 y, she developed photophobia and visual disturbances; bilateral cataracts were diagnosed shortly after (Table 2). CME was diagnosed at 21 y. She underwent cataract surgery (RE) at 25 y. At her most recent evaluation, BCVA was 20/80 (RE) and 20/100 (LE). Multimodal retinal imaging revealed diffuse RPE dystrophy, consistent with an atypical form of RP (Fig. 2g–i). Microperimetry showed a relatively stable fixation with reduced mean sensitivity. GVF was constricted to 30° centrally. ffERG demonstrated severe rod-cone dysfunction with extinguished scotopic and subnormal photopic responses.

Disease progression was evident from the longitudinal data of the 10-year follow-up. Specifically, BCVA showed an early decline from 0.6 to 0.3 during her teenage years and remained stable for a decade before dropping to 0.2. Microperimetry values were low but stable throughout her 20 s, before slowly declining. ffERG showed a steady decline with a reduction in photopic response amplitude (average progression rate: −3.1 µV/year).

##### Molecular findings in P3

Exome Sequencing (ES) identified a homozygous nonsense variant in exon 3 of *RDH11* (M2, Fig. 1b,h). The variant (NM_016026.4:c.235G>T, p.Glu79Ter) is predicted to introduce a PTC at amino acid position 79, has an AF of 6.8 × 10^−7^ in gnomAD, and is classified as pathogenic according to the ACMG criteria (PVS1_v.strong/PM2_supporting/PM3_supporting/PP3_supporting/PP4_supporting) (Table 3). Mutant transcripts are predicted to undergo NMD. The variant was recently described at homozygous state in an affected subject (Table 3) [10].

#### Family 3: patient P4

##### Clinical findings in P4

Patient P4, a Caucasian male, presented to the eye clinic at the age of 58 y. He was diagnosed with RP at 35 y and subjectively reported night blindness, glare sensitivity and reduced contrast vision. He had bilateral cataract surgery at the age of 6-7 y with subsequent aphakia, corrected with contact lenses. Additionally, he suffered a retinal detachment in his RE at the age of 36 y, treated with vitrectomy.

The patient was born to consanguineous parents and had an unaffected sister (Fig. 1c). He suffered from microcephaly with reported spasms in childhood. Psychomotor development was delayed with reduced mental state, for which his mother served as legal representative. At the age of 57 y, he had a pulmonal emboly of unknown aetiology (Table 1).

Ophthalmological examination revealed blindness in RE and reduced BCVA (20/80) in LE (Table 2). Besides aphakia, the RE had remnants of the intraocular silicone oil in the anterior chamber and presented with corneal neovascularisation. Insight into the posterior eye pole was reduced, with atrophic retina and optic nerve head, and attenuated vessels (Fig. 2j, k). The LE exhibited similar signs of advanced retinal degeneration with epiretinal membrane and cystic retinoschisis in the macula (Fig. 2l). The visual field was not measurable in the RE and severely concentrically reduced in the LE. Dark-adaptation thresholds were pathologically elevated and mixed (cone/rod-mediated).

##### Molecular findings for P4

GS identified a homozygous nonsense variant (NM_016026.4:c.749G>A; p.Trp250Ter) in *RDH11* (M3, Fig. 1c,h) predicted to introduce a PTC at amino acid 250. Mutant transcripts are predicted to undergo NMD. The variant is classified as pathogenic according to the ACMG criteria (PVS1_v.strong/PM2_supporting/PM3_supporting/PP3_supporting/PP4_supporting), has an AF of 0.0007% in gnomAD and has not yet been reported in literature (Table 3). In addition, a heterozygous variant of uncertain significance (VUS) was detected in *SNRNP200* (NM_014014.5:c.2003A>C;p.Asp668Ala), with an AF of 0.0001% in gnomAD. No samples for segregation analysis have been available.

#### Family 4: patient P5

##### Clinical findings in P5

P5 is a male patient who presented to the eye clinic at 33 y due to reduced vision, reporting night blindness predominantly. He had cataract surgery in both eyes at the age of 5 y with subsequent pseudophakia and glasses correction.

The patient was born preterm to non-consanguineous parents and had two unaffected siblings (Fig. 1d). The family history was unremarkable. He suffered from developmental delay, for which his mother served as legal representative, and a coagulation disorder causing repeated thromboses. Additionally, the patient had hepatitis B (Table 1).

Ophthalmological examination revealed a moderately reduced visual acuity (20/50 RE, 20/40 LE) (Table 2). Both eyes had pseudophakia with iridectomies. The visual field was only slightly concentrically reduced. ffERG scotopic and photopic responses were non-recordable. Dark-adaptation thresholds, measured with a full-field stimulus threshold, were pathologically elevated and cone-mediated. Colour test revealed a blue colour perception pathology, typical for early-stage retinal dystrophies (Table 2). Retinal imaging showed a generalised degeneration with narrow vessels (Fig. 2m), and a more pronounced atrophy in the central region and periphery, as seen in FAF (Fig. 2n). Still, small remnants of foveal photoreceptor layering were visible in both eyes by SD-OCT (Fig. 2o).

##### Molecular findings for P5

GS identified a homozygous 2-bp insertion variant (NM_016026.4:c.688_689insGC; p.Val230GlyfsTer18) in *RDH11* (M4, Fig. 1d,h) resulting in a frameshift and PTC, predicted to induce NMD of the mutant transcript. This variant is classified as pathogenic according to the ACMG criteria (PVS1_v.strong/PM2_supporting/PM3_supporting/PP4_supporting), and has neither been observed in gnomAD nor in the literature (Table 3). In addition, single heterozygous variants in *RIMS1* (NM_014989.7:c.3430C>T;p.Arg1144Ter) and *PDE6B* (NM_000283.4:c.1935C>A;p.Tyr645Ter) were observed. As both genes are associated with autosomal recessive inheritance, they are not expected to be disease-causing in this patient.

#### Family 5: patient 6

##### Clinical findings in P6

P6 is a male child born to reportedly non-consanguineous Black British parents. He is an only child and there is no relevant family history of note (Fig. 1e). He presented prenatally from 28-week gestation with intrauterine growth restriction and was born at term with a birth weight -3 standard deviations (SD) for age/sex. He was diagnosed with sickle cell anaemia (mother HbAC, father HbAS carriers). His growth parameters fell further with age and at 6 y height, weight, and occipito-frontal circumference measured approximately -4, -5, and -6SD, respectively. His growth hormone level testing (Insulin-Like Growth Factor-1) results were equivocal and parents opted out of a growth hormone treatment. His imaging was not suggestive of a skeletal dysplasia. His postnatal course has been notable for global developmental delay, intellectual disability, autism, attention-deficit/hyperactivity disorder (ADHD), and a history of seizures that have since resolved (Table 1). At 9-10 y, his learning was felt to be delayed by around 3-4 y. Brain MRI demonstrated bilateral subcortical white-matter signal abnormalities, thinning of the corpus callosum, and progressive cerebellar volume loss. There was also a suggestion of iron deposition in the globi pallidi. The pituitary gland was normal.

P6 was diagnosed with bilateral zonular cataracts and visual impairment at the age of 6 y, and subsequently underwent cataract surgery, resulting in bilateral pseudophakia (Table 2). Despite a technically successful surgery, his vision failed to improve to the degree expected. No overt fundal changes were noted on examination (Fig. 2p–r), though the retina did have a slightly dark/grey appearance. ffERG testing indicated a generalised retinal dysfunction, with greater involvement of rod vs cone pathways. Pattern reversal visual evoked potentials demonstrated bilateral macular pathway dysfunction with some preservation of macular function.

##### Molecular findings in P6

Trio GS identified a homozygous missense variant in *RDH11*, c.455G>A; p.(Gly152Asp) (M5, Fig. 1e,h) which affects the acceptor splice consensus sequence of exon 2 and is classified as likely pathogenic according to ACMG criteria (PM2_supporting/PM3_supporting/PP3_v.strong/PP4_supporting) and prediction tools (CADD score = 34; REVEL = 0.918; MutScore = 0.890) [12–14] (Table 3). In silico analyses predict that the Gly152 residue is intolerant to variation (Supplementary Fig. 2). Moreover, the substitution of a small and flexible amino acid (glycine) with the larger and charged aspartic acid is predicted to destabilise RDH11 (Supplementary Fig. 3). Unaffected parents were heterozygous carriers, confirming biallelic inheritance (Fig. 1e).

#### Family 6: patient 7

##### Clinical findings in P7

Patient P7 is an 11-year-old South Asian male, born to consanguineous parents with an otherwise unremarkable family history (Fig. 1f). He was born at term following a normal vaginal delivery (birth weight: 2580 g) and presented with congenital microcephaly and subtle dysmorphic features. His growth trajectory has been characterised by disproportionate short stature and failure to thrive. He has global developmental delay and mild-to-moderate intellectual impairment, with very poor school performance. Craniofacial evaluation revealed the presence of craniosynostosis, sloping forehead, abnormality of the maxilla, abnormal facial shape, and abnormal dentition (Table 1). The overall clinical course has been stable over time, without significant deterioration or improvement.

He was first seen at an eye clinic at the age of 7 y for bilateral poor vision and strabismus and was diagnosed with RP and blue dot cataracts. He has been wearing spectacles since then. An orthoptic examination performed at the age of 10 y showed a non-specific intermittent distance exotropia with slow fixation, poor stereopsis, and reduced near point of convergence (10 cm). Ocular motility examination revealed gaze-evoked nystagmus with mild bilateral inferior oblique overaction and superior rectus underaction (±1), and a V-pattern exotropia (Table 2). Multimodal imaging revealed retinal dystrophy with subtle RPE mottling, vascular attenuation, and mild thinning of the outer retinal layers (Fig. 2s–u).

##### Molecular findings in P7

GS identified a homozygous frameshift deletion in exon 5 of *RDH11* (NM_016026.4:c.601_608del; p.Ala201SerfsTer35), predicted to introduce a PTC (M6, Fig. 1f,h) with transcripts undergoing NMD. The variant is novel, absent from gnomAD and classified as pathogenic based on ACMG criteria (PVS1_v.strong/PM2_supporting/PM3_supporting/PP4_supporting) (Table 3). This patient was diagnosed through an international effort at the 2024 Undiagnosed Hackathon [15].

#### Family 7: patients 8-13

Family 7 comprises six affected siblings (P8-P13) out of 10 born to consanguineous parents (Fig. 1g). All affected individuals have a history of cataracts, seizures and mild to severe intellectual disability. ES of P8 identified a homozygous missense variant (NM_016026.4:c.413C>T, p.Thr138Ile) in *RDH11* (M7, Fig. 1g,h), classified as likely pathogenic based on the ACMG criteria (PM2_supporting/PM3_supporting/PP1_supporting/PP3_supporting/PP4_supporting) (Table 3). The affected sequence is highly conserved and the amino acid exchange is predicted to be deleterious (CADD score = 31; REVEL = 0.704; MutScore = 0.804) [12–14]. Supporting this, in silico structural assessments predict that the Thr138 residue is intolerant to variation and that substituting this polar amino acid with a non-polar isoleucine destabilises RDH11 (Supplementary Figs. 2 and 3). Segregation analysis showed that all affected siblings carry the same homozygous missense variant, while the parents and one unaffected sibling are heterozygous carriers (Fig. 1g). No further rare variants in genes potentially responsible for overlapping phenotypes were observed. Although the depth of clinical phenotyping for Family 7 was restricted by geopolitical circumstances and barriers, the proper segregation of the novel variant across all analysed family members (i.e. the proband, five affected siblings, an unaffected sibling, and both parents) and their consistent clinical presentation, which included key manifestations such as cataracts, seizures, and variable degrees of intellectual disability, render this family highly informative to the overall genetic and phenotypic spectrum.

## Discussion

The *RDH11*–disease association is provisional in Online Mendelian Inheritance in Man (OMIM #607849; March 2026). Consistently, RetiGene [16] classifies *RDH11* as a candidate rather than an established IRD gene. Here, we present a large multi-center cohort with *RDH11*-associated disease, comprising eight individuals from seven unrelated families, with comprehensive clinical and genetic characterisation. These findings strengthen the evidence for a causal role of biallelic *RDH11* variants in a consistent syndromic phenotype marked by juvenile bilateral cataracts, rod-cone dysfunction, intellectual disability, and growth/neurodevelopmental features. In addition, our findings: (i) expand the mutational spectrum of *RDH11*, (ii) corroborate the association proposed for *RDH11* [7–10], and (iii) help resolve the uncertain OMIM gene-disease association for *RDH11* supporting its recognition as a bona fide IRD gene.

**Fig. 3:**
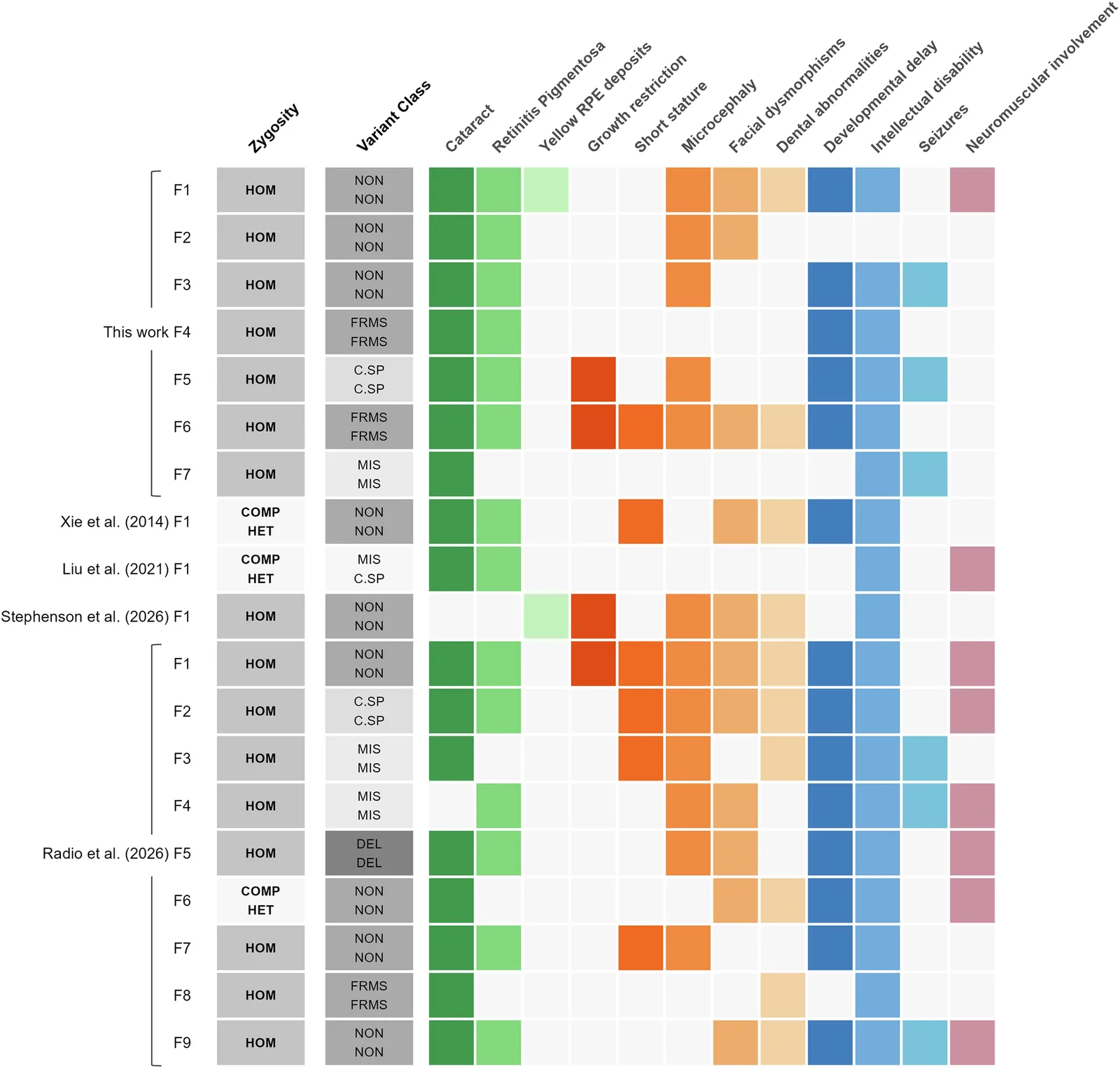
Overview of the phenotypic and genetic features of subjects with *RDH11*-related disease. The heatmap summarises the genotypic characteristics (zygosity and variant functional class) and clinical phenotyping data for the seven families described in this study (F1–F7), alongside previously reported families [7–10]. Phenotypic categories are colour-coded as follows: ophthalmological features in shades of green, growth and craniofacial dysmorphisms in orange, neurodevelopmental traits in blue, and muscular involvement in purple. Solid coloured boxes denote the presence of a specific clinical feature, whereas blank boxes indicate its absence. In the genotype columns, dark grey shading highlights individuals harbouring biallelic bona fide truncating variants (nonsense or frameshift). Note that the homozygous missense variant identified in F5 affects the first nucleotide of exon 2 and thereby the splicing junction sequence (‘C.SP’), and is predicted to disrupt normal splicing. C.SP splicing consensus variant, COMP HET compound heterozygous, FRMS frameshift, HOM homozygous, MIS missense, NON nonsense.

All affected individuals in our cohort (8/8; Table 2, and Fig. 3) presented with bilateral congenital or early-childhood cataracts, requiring surgical intervention. Visual impairment from these cataracts was the initial sign prompting ophthalmological assessment. Unlike typical retinal dystrophies, where cataracts develop as late secondary complications, in *RDH11*-related disease, early-onset juvenile cataracts often precede retinal symptoms, a finding that deserves further investigation. Subsequent examination revealed a rod-cone dystrophy in 7/8 subjects, with typical RP features, such as vascular attenuation, diffuse RPE dystrophy, and variable pigment deposits in the mid-periphery. OCT confirmed outer retinal thinning and severe EZ band disruption. In three of these seven subjects (42.8%), the retinopathy was complicated by CME, a finding commonly observed in both isolated and syndromic forms of RP [17]. In terms of photoreceptor function, ERG findings from six individuals showed severely compromised responses, predominantly in the scotopic component, indicating primary rod involvement, consistent with the referred night blindness and progressive mid-peripheral visual field loss. Concomitant impairment of central visual acuity and photopic responses in all subjects suggested secondary cone involvement.

In one patient (P1), we observed numerous yellowish RPE deposits, localised mainly in mid-periphery (Supplementary Fig. 1). These findings parallel those described in a patient homozygous for the nonsense variant c.216C>A; p.Cys72Ter [9]. Differently from P1, that patient had remained asymptomatic in terms of visual function and exhibited a clear lens and normal ERG responses (at least until the time of clinical assessment at the age of 7 y), despite irregularities in the RPE and EZ reflectivity. His extraocular features (such as developmental delay, dental anomalies, microcephaly, and dysmorphisms) were nevertheless overall consistent with previously described *RDH11*-phenotypes [7–9]. The authors termed this distinct phenotype “retinal pigment epitheliopathy due to suboptimal recycling of vitamin A” and hypothesised that the milder presentation may result from transcriptional adaptation and compensation from other RDH family members.

Regarding extra-ocular involvement, our cohort exhibited a high prevalence of neurodevelopmental disorders (7/8 subjects), including intellectual disability, autism spectrum disorder, cognitive delay, learning difficulties, language skill impairment, and seizures (3/8 subjects) (Table 1). Consistent with previous reports [7–9], common features included congenital microcephaly (6/8 subjects), intrauterine and postnatal growth restriction (3/8 subjects), and facial dysmorphisms (e.g., micrognathia, midface hypoplasia) combined with dental abnormalities (2/8 subjects) (Fig. 3). Notably, one individual (P1) presented with myopathy signs, supporting recent evidence that skeletal muscle involvement expands the biallelic *RDH11* phenotypic spectrum [8,10]. However, systematic neuromuscular assessments were not performed as the cohort was recruited through ophthalmology clinics. Except for P1, no patients reported overt symptoms (e.g., weakness or fatigue); thus, advanced evaluations, like imaging or biopsy, were not clinically indicated. Because *RDH11*-associated myopathy can be mild, progressive, or subclinical early on [10], an underlying involvement cannot be definitively ruled out in the remaining patients. Future longitudinal studies with targeted screening are necessary to fully determine myopathy prevalence.

While *RDH11*-related retinal phenotypes stem from visual cycle disruptions, its extraretinal features highlight the systemic importance of vitamin A metabolism. *RDH11* encodes an enzyme vital for synthesising retinoic acid (RA), an active vitamin A metabolite that regulates gene expression during embryonic development and tissue maintenance [18]. Precise RA gradients are essential for neural tube patterning, neurogenesis, and neural crest cell migration [19]; disrupting these pathways likely causes the microcephaly, neurodevelopmental delays, and distinct craniofacial features seen in patients. Additionally, because RA signaling regulates myoblast differentiation and skeletal muscle homeostasis [20–22], its disruption offers a plausible molecular basis for the progressive myopathy associated with systemic RDH11 loss.

The limited number of subjects with *RDH11*-related disease reported in the literature likely reflects the infrequent occurrence of pathogenic variants in this gene. Based on the estimated carrier frequencies [23], the expected number of *RDH11*-associated patients in the European (non-Finnish) population is significantly lower compared to other rare IRDs (approximately 58-fold less than *RDH12*-related conditions, 93-fold lower than anticipated *RDH5* patients, and 118-fold lower than *RPE65*). These findings are consistent with our cohort, in which all eight described subjects were homozygous for the causative *RDH11* variant (Table 1, and Fig. 3). This suggests a disease arising, at least in part, from founder effects or, as observed here, from high rates of consanguinity (families 3, 6, 7) and extensive regions of homozygosity (family 1). Overall, we identified seven different *RDH11* variants, two of which are novel (not in the literature or ClinVar; Table 3). These include five truncating variants (3 nonsense, 2 frameshift), and two missense alleles (one involving a splice junction sequence), strengthening loss-of-function as the primary pathogenic mechanism in *RDH11*-associated retinopathy. Pathogenicity predictions and in silico protein modelling assessments strongly support a disruptive effect of the two missense variants on RDH11 function (Supplementary Figs. 2 and 3), yet experimental functional evidence is warranted to unequivocally validate this deleterious effect.

Our findings provide an independent replication of the role of *RDH11* in syndromic retinal disease. To delineate the phenotypic spectrum associated with *RDH11* mutations, we performed a systematic comparison of our cohort with previously reported patients, including the cohort of Radio et al. which was published concurrently with our submission [7–10] (Fig. 3). Through this comparison, we demonstrate that biallelic *RDH11* variants are almost invariably associated with a multisystem phenotype that includes bilateral congenital or early-childhood cataracts, RP, failure to thrive, and developmental delay with varying degrees of intellectual disability. These features are frequently accompanied by microcephaly, facial dysmorphisms, and dental anomalies, largely recapitulating the clinical spectrum first described by Xie et al. [7] (Fig. 3). Although this constitutes the most recurrent phenotype, biallelic *RDH11* genotypes may present with additional clinical manifestations, such as mild myopathy [8, 10] or, conversely, with normal visual function and clear lenses [9]. The identified pleiotropic phenotypes necessitate detailed clinical characterisation of larger cohorts to delineate the full *RDH11-*associated disease spectrum, elucidate genotype-phenotype correlations, and investigate whether compensatory transcriptional activation underlies milder manifestations.

## Supplementary information

Supplementary Information

## Data Availability

The identified variants have been deposited in LOVD v.3.0 (Leiden Open Variation Database; http://www.lovd.nl/). Depersonalised datasets analysed in the current study are available from the authors on reasonable request. Data from the National Genomic Research Library (NGRL) used in this research are available within the secure Genomics England Research Environment. Access to NGRL data is restricted to adhere to consent requirements and protect participant privacy. Data used in this research include variant data from participants recruited via the NHS Genomic Medicine Service (located in the /genomes/by_date folder), including rare disease probands and family members. Access to NGRL data is provided to approved researchers who are members of the Genomics England Research Network, subject to institutional access agreements and research project approval under participant-led governance. For more information on data access, visit: http://www.genomicsengland.co.uk/research.

## References

[1] Sahu B, Maeda A. Retinol dehydrogenases regulate vitamin A metabolism for visual function. Nutrients. 2016;8:746. 10.3390/nu8110746. PMC5133129 27879662

[2] Sajovic J, Meglič A, Glavač D, Markelj Š, Hawlina M, Fakin A. The role of vitamin A in retinal diseases. IJMS. 2022;23:1014. 10.3390/ijms23031014. PMC8835581 35162940

[3] Haeseleer F, Jang GF, Imanishi Y, Driessen CAGG, Matsumura M, Nelson PS, et al. Dual-substrate specificity short chain retinol dehydrogenases from the vertebrate retina. J Biol Chem. 2002;277:45537–46. 10.1074/jbc.M208882200. PMC1435693 12226107

[4] Lin B, White JT, Ferguson C, Wang S, Vessella R, Bumgarner R, et al. Prostate short-chain dehydrogenase reductase 1 (PSDR1): a new member of the short-chain steroid dehydrogenase/reductase family highly expressed in normal and neoplastic prostate epithelium. Cancer Res. 2001;61:1611–8. 11245473

[5] Belyaeva OV, Wu L, Shmarakov I, Nelson PS, Kedishvili NY. Retinol dehydrogenase 11 is essential for the maintenance of retinol homeostasis in liver and testis in mice. J Biol Chem. 2018;293:6996–7007. 10.1074/jbc.RA117.001646. PMC5936824 29567832

[6] Kanan Y, Wicker LD, Al-Ubaidi MR, Mandal NA, Kasus-Jacobi A. Retinol dehydrogenases RDH11 and RDH12 in the mouse retina: expression levels during development and regulation by oxidative stress. Invest Ophthalmol Vis Sci. 2008;49:1071–8. 10.1167/iovs.07-1207. PMC2366097 18326732

[7] Xie YA, Lee W, Cai C, Gambin T, Nõupuu K, Sujirakul T, et al. New syndrome with retinitis pigmentosa is caused by nonsense mutations in retinol dehydrogenase RDH11. Hum Mol Genet. 2014;23:5774–80. 10.1093/hmg/ddu291. PMC4189905 24916380

[8] Liu YD, Huang SS, Li M, Lek M, Song DY, Tan DD, et al. A new phenotype of syndromic retinitis pigmentosa with myopathy is caused by mutations in retinol dehydrogenase 11. Clin Genet. 2022;101:448–53. 10.1111/cge.14108. 34988992

[9] Stephenson KAJ, Shao Z, Tumber A, Tavares E, Ahmed K, Higginbotham EJ, et al. Retinal pigment epitheliopathy due to sub-optimal recycling of vitamin A (RESORVA): a novel RDH11-related phenotype. Clin Genet. 2025. 10.1111/cge.70128. PMC13066773 41459630

[10] Radio FC, Tasca G, Coppens S, Chillemi G, Whalen S, Marey I, et al. Loss of function of retinol dehydrogenase 11 causes a recessive syndrome characterized by myopathy, retinal dystrophy, juvenile cataracts, and microcephaly. Genet Med. 2026;28:102558. 10.1016/j.gim.2026.102558. 41904678

[11] Hamel C. Retinitis pigmentosa. Orphanet J Rare Dis. 2006;1:40. 10.1186/1750-1172-1-40. PMC1621055 17032466

[12] Rentzsch P, Witten D, Cooper GM, Shendure J, Kircher M. CADD: predicting the deleteriousness of variants throughout the human genome. Nucleic Acids Res. 2019;47:D886–94. 10.1093/nar/gky1016. PMC6323892 30371827

[13] Ioannidis NM, Rothstein JH, Pejaver V, Middha S, McDonnell SK, Baheti S, et al. REVEL: an ensemble method for predicting the pathogenicity of rare missense variants. Am J Hum Genet. 2016;99:877–85. 10.1016/j.ajhg.2016.08.016. PMC5065685 27666373

[14] Quinodoz M, Peter VG, Cisarova K, Royer-Bertrand B, Stenson PD, Cooper DN, et al. Analysis of missense variants in the human genome reveals widespread gene-specific clustering and improves prediction of pathogenicity. Am J Hum Genet. 2022;109:457–70. 10.1016/j.ajhg.2022.01.006. PMC8948164 35120630

[15] Delgado-Vega AM, Cederroth H, Taylan F, Ekholm K, Ek M, Thonberg H, et al. Pushing the boundaries of rare disease diagnostics with the help of the first Undiagnosed Hackathon. Nat Genet. 2024;56:2287–94. 10.1038/s41588-024-01941-1. PMC12198426 39433890

[16] Rivolta C, Celik E, Kamdar D, Cancellieri F, Kaminska K, Ullah M, et al. RetiGene, a comprehensive gene atlas for inherited retinal diseases. Am J Hum Genet. 2025;112:2253–65. 10.1016/j.ajhg.2025.08.017. PMC12696501 40961941

[17] Testa F, Karali M, Boccia R, Pisani D, Damiano L, Nicolò A, et al. Cystoid macular edema in non-syndromic retinitis pigmentosa: associations with causative genes in a large cohort. Invest Ophthalmol Vis Sci. 2025;66:5. 10.1167/iovs.66.12.5. PMC12410279 40900079

[18] Niederreither K, Dollé P. Retinoic acid in development: towards an integrated view. Nat Rev Genet. 2008;9:541–53. 10.1038/nrg2340. 18542081

[19] Martínez-Morales PL, Diez del Corral R, Olivera-Martínez I, Quiroga AC, Das RM, Barbas JA, et al. FGF and retinoic acid activity gradients control the timing of neural crest cell emigration in the trunk. J Cell Biol. 2011;194:489–503. 10.1083/jcb.201011077. PMC3153641 21807879

[20] Ryan T, Liu J, Chu A, Wang L, Blais A, Skerjanc IS. Retinoic acid enhances skeletal myogenesis in human embryonic stem cells by expanding the premyogenic progenitor population. Stem Cell Rev Rep. 2012;8:482–93. 10.1007/s12015-011-9284-0. 21735106

[21] Lamarche É, Lala-Tabbert N, Gunanayagam A, St-Louis C, Wiper-Bergeron N. Retinoic acid promotes myogenesis in myoblasts by antagonizing transforming growth factor-beta signaling via C/EBPβ. Skelet Muscle. 2015;5:8. 10.1186/s13395-015-0032-z. PMC4397812 25878769

[22] Kennedy KAM, Porter T, Mehta V, Ryan SD, Price F, Peshdary V, et al. Retinoic acid enhances skeletal muscle progenitor formation and bypasses inhibition by bone morphogenetic protein 4 but not dominant negative beta-catenin. BMC Biol. 2009;7:67. 10.1186/1741-7007-7-67. PMC2764571 19814781

[23] Hanany M, Rivolta C, Sharon D. Worldwide carrier frequency and genetic prevalence of autosomal recessive inherited retinal diseases. Proc Natl Acad Sci USA. 2020;117:2710–6. 10.1073/pnas.1913179117. PMC7007541 31964843

