# Supplementary material for: Biallelic RDH11 variants cause syndromic retinitis pigmentosa with early-onset cataracts and neurodevelopmental delay: a multicenter case series
Source: Eur J Hum Genet. 2026 Sep 16;34(11):1663–75. doi: 10.1038/s41431-026-02232-5 (PMC13743764; doi:10.1038/s41431-026-02232-5)
Supplement: Supplementary file 1 [file 41431_2026_2232_MOESM1_ESM.pdf]

Biallelic *RDH11* variants cause syndromic Retinitis pigmentosa with early-onset cataracts and neurodevelopmental delay: a multicenter case series

Marianthi Karali, Susanne Kohl, Francesco Testa, Eleanor Hay, Yasas D. Kolambage, German Demidov, Tobias Haack, Hamid Ghaedi, Siying Lin, Gemma Natale, Vindya Subasinghe, Pyara Rathnayake, Sarah Wörz, Gavin Arno, Rebecca Buchert, Katarina Stingl, Francesca Simonelli, Sandro Banfi

Supplementary Information

- Supplementary Figure 1. Yellow RPE deposits at the mid-peripheral retina in patient P1.
- Supplementary Figure 2. *In silico* assessment of the pathogenicity of the *RDH11* missense variants.
- Supplementary Figure 3. DynaMut2 homology modelling of the predicted structural impact and stability of *RDH11* missense variants.
- Supplementary File 1. Supplementary Information on Subjects and Methods
- Supplementary References

Supplementary Figure 1

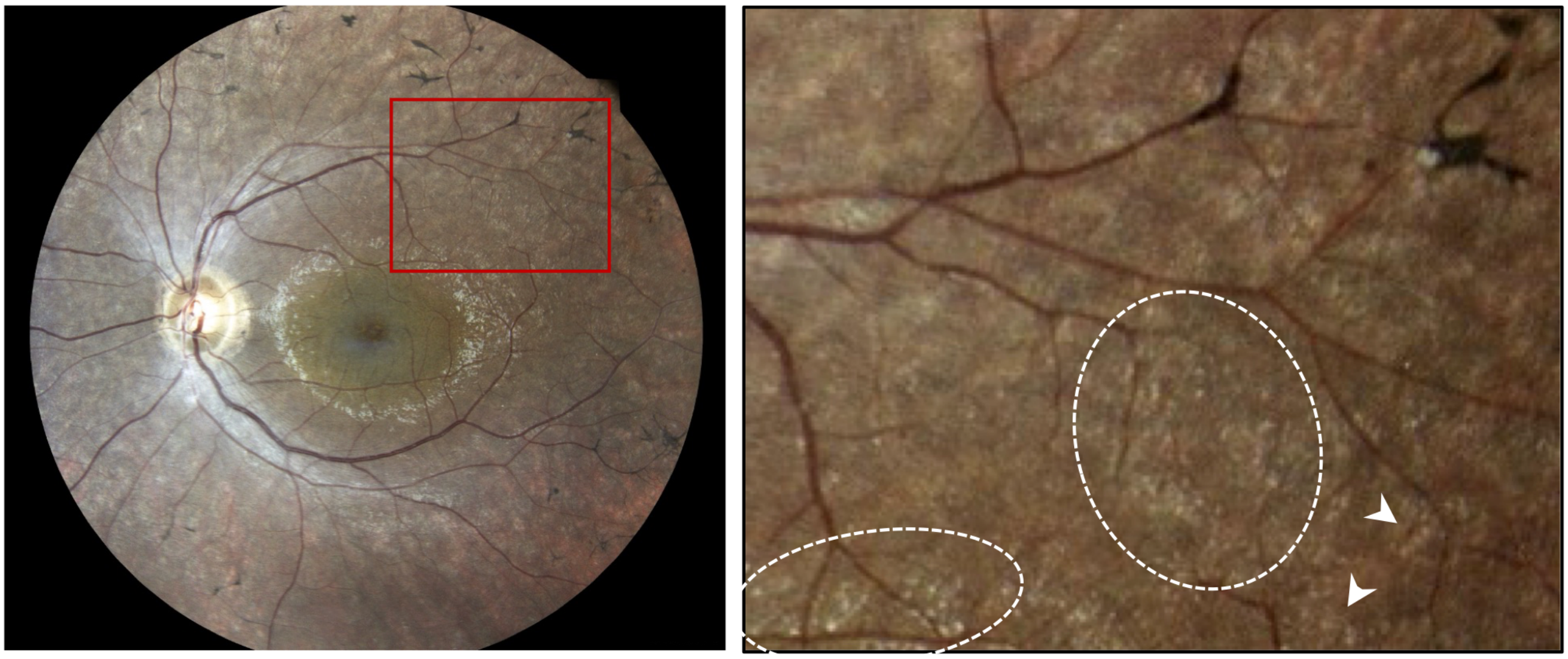

Supplementary Figure 1. Yellow RPE deposits at the mid-peripheral retina in patient P1.

(*left*) Fundus photograph of the eye of P1 illustrating yellow subretinal RPE deposits.

(*right*) Magnified view of the region demarcated by the red box in the fundus photograph, highlighting numerous yellow deposits in the mid-periphery (white arrowheads and dashed circles).

Supplementary Figure 2

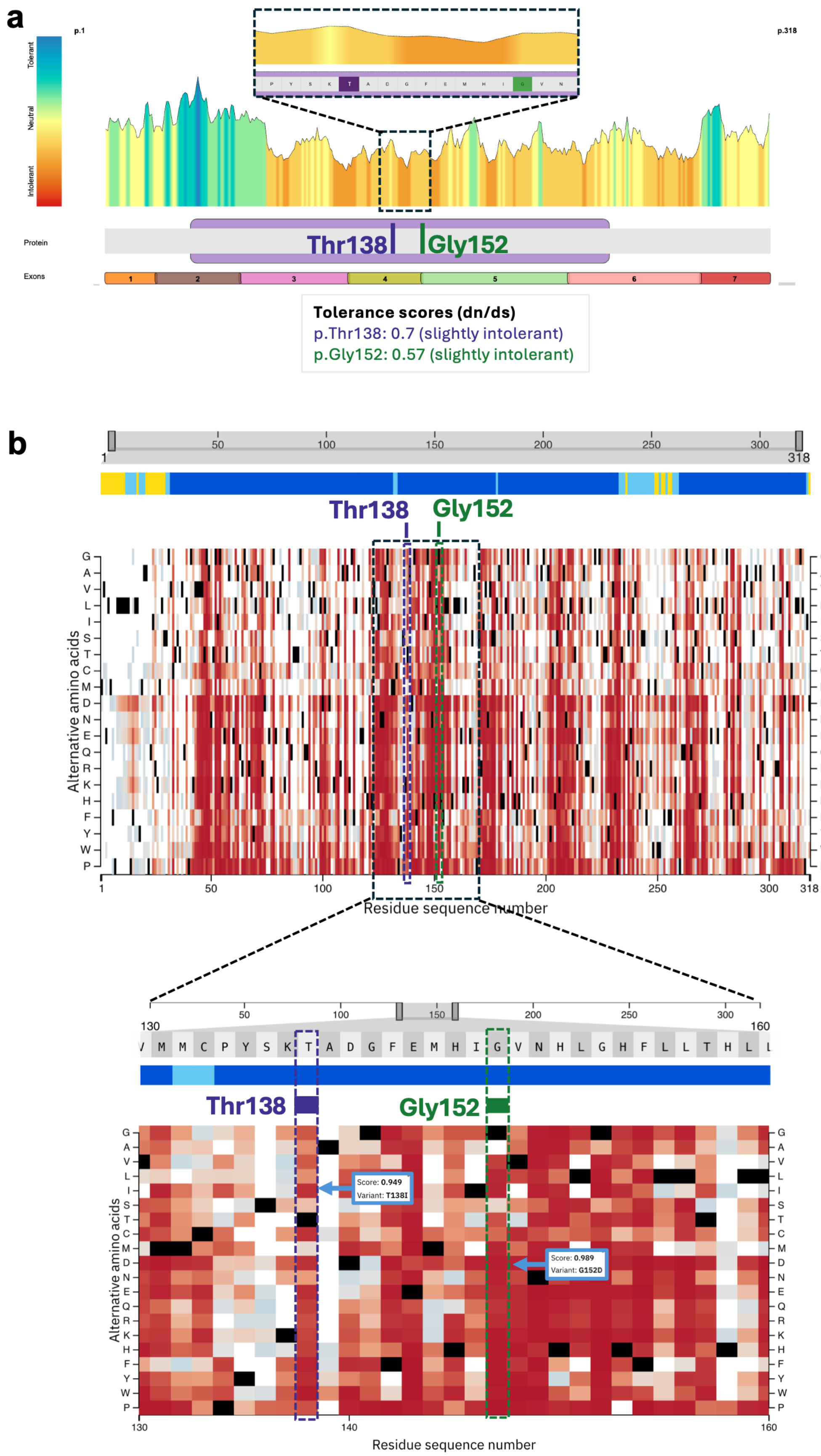

Supplementary Figure 2. *In silico* assessment of the pathogenicity of the *RDH11* missense variants.

(**a**) Tolerance landscape visualization of the human *RDH11* gene (NM_016026.3) generated via the MetaDome 2.0 web-server (http://www.metadome.app/metadome/)(1). Vertical lines in the protein structure diagram indicate the positions of the amino acid residues (Thr138 in exon 4; Gly152 in exon 5) affected by the missense variants identified in this cohort. The corresponding region is enlarged within the dashed-line box. The tolerance scores (0.70 for Thr138; 0.57 for Gly152) indicate that both residues are slightly intolerant to variation.

(**b**) AlphaMissense (2) pathogenicity scores for the predicted p.Thr138Ile (0.949) and p.Gly152Asp (0.989) substitutions, indicating that these variants are likely to disrupt RDH11 function. The inset shows a magnified view of the region spanning both residues, displaying the color-coded pathogenicity scores for all possible amino acid substitutions at positions Thr138 and Gly152.

Supplementary Figure 3

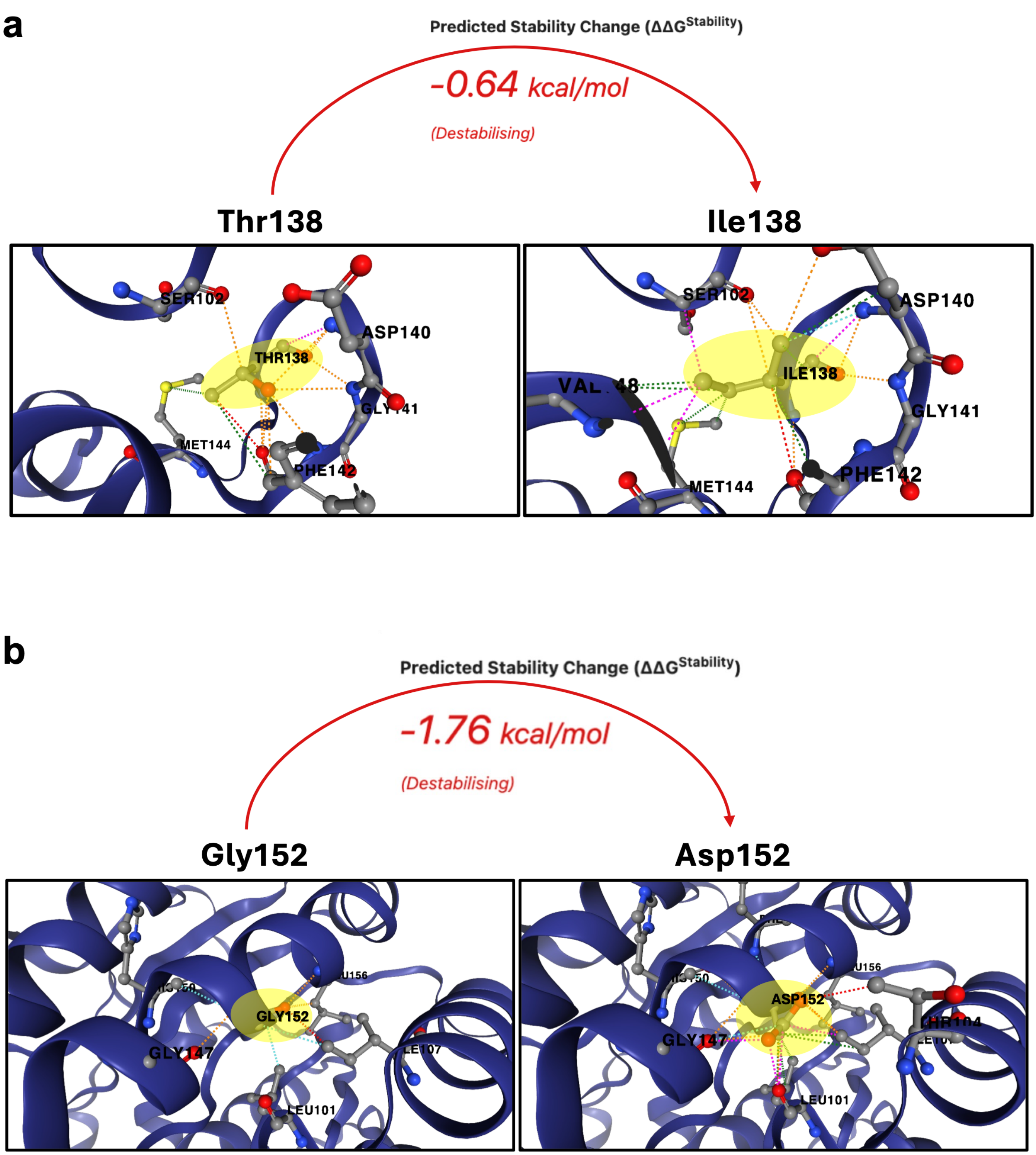

Supplementary Figure 3. DynaMut2 homology modelling of the predicted structural impact and stability of *RDH11* missense variants.

The p.Thr138Ile **(a)** and p.Gly152Asp **(b)** substitutions in RDH11 (AF-Q8TC12-F1-model_v6.pdb) are predicted by DynaMut2 (3) to destabilise the protein structure, with predicted free energy changes ($\Delta\Delta G$) of -0.64 kcal/mol and -1.76 kcal/mol, respectively. The Thr138Ile variant replaces the polar threonine with the non-polar amino-acid, whereas the Gly152Asp substitutes a small and flexible amino acid with the larger and charged aspartic acid. Both variants are predicted to disrupt proper bonding with spatially adjacent amino acid residues and induce intramolecular clashes. Dotted lines indicate hydrogen bonds (red), hydrophobic interactions (green), and polar interactions (orange). Pink dashed lines denote intramolecular clashes that could potentially compromise protein stability.

Supplementary File 1

Supplementary Information on Subjects and Methods

Subjects

Subjects were diagnosed at the Referral Centre for Inherited Retinopathies of the University of Campania 'Luigi Vanvitelli', Italy (patients P1-P3), the Centre for Ophthalmology of the University of Tübingen, Germany (P4, P5), the Great Ormond Street Hospital, UK (P6), the Lady Ridgeway Hospital for Children, Sri Lanka (P7), and the Medical Genetics Unit, Shahid Beheshti University of Medical Sciences, Tehran, Iran (P8). Implemented procedures adhered to the tenets of the Declaration of Helsinki and were approved by the Ethics Committees of the participating institutes. A comprehensive medical history was collected for all subjects including basic auxiological data. Peripheral blood samples were collected upon written informed consent of the subjects or their legal guardians to genetic analysis.

Ophthalmological examination

Subjects underwent a complete ophthalmological examination which included best-corrected visual acuity (BCVA) measurements, slit lamp of anterior segment, intraocular pressure, fundus examination, kinetic visual field (KVF), spectral domain optical coherence tomography (SD-OCT), fundus autofluorescence imaging (FAF), standard full-field electroretinography (ERG), microperimetry (for P3) and full-field stimulus test (FST) (for P4 and P5). KVF was measured with the Octopus 900 device (Haag-Streit, Switzerland) or by moving the III4e and V4e stimulus target on a calibrated standard Goldmann perimeter. SD-OCT and FAF were performed with a Heidelberg Eye Explorer Version 1.9.11.0 (Heidelberg, Germany) and the California P200DTx (Optos, Dunfermline, UK). FST was performed using the Espion systems with blue and red stimuli (Diagnosys, Lowell, MA, USA) and 0 dB set to 0.01 cd.s/m2. ERG was performed following pupil dilation using 1% tropicamide and 2.5% phenylephrine hydrochloride, according to International Society for Clinical Electrophysiology of Vision (ISCEV) standards using an Espion Visual Electrophysiology System (Diagnosys Vision Ltd, Dublin, Ireland). The protocol included rod-specific and standard bright flash ERG, both recorded after a minimum of 20 minutes dark adaptation.

Sequencing and variant prioritisation approach

Genomic DNA was extracted from peripheral blood according to standard protocols. Subjects were analysed either by Exome Sequencing (ES; for P3 and P8) or Genome Sequencing (GS; for P1, P4, P5, P6, P7). P2 and affected family 7 members were analysed by Sanger-based search of the variants identified in affected siblings (in P1 and P8, respectively). ES and GS libraries were prepared using the SureSelect Human All Exon V7 (Agilent Technologies) and the llumina DNA PCR-Free Library Prep (Illumina, San Diego, CA, USA), as described previously (4). The called single nucleotide variants (SNV) were annotated as previously described (5). Only variants with a minor allele frequency (MAF) < 0.05 in the Genome Aggregation Database (gnomAD; http://gnomad.broadinstitute.org) were retained. Variant classification was performed according to the American College of Medical Genetics and Genomics (ACMG) guidelines using Varsome (http://varsome.com/). The Emedgene™ software (Illumina inc., San Diego, CA, USA) was used for variant prioritisation in P1 and P3. The variants were validated by Sanger sequencing of the corresponding genomic fragments. Segregation analysis of the identified variants was performed whenever samples from additional family members were available. For P6, trio GS (proband and unaffected parents) was performed through the NHS Genomic Medicine Service (GMS), with clinical pipeline analysis focusing on protein-altering variants, including single nucleotide variants (SNVs), short insertion-deletion variants (INDELs) and structural variants (SVs) and copy number variants (CNVs), using DRAGEN pipelines. Variant interpretation was performed within these applied Genomics England PanelApp virtual gene panels (available at https://panelapp.genomicsengland.co.uk/) tailored to the individual's phenotype: "Paediatric disorders" panel v35.2, "Bilateral congenital or childhood onset cataracts" panel v4.0, "Severe microcephaly" panel v4.0, "Cerebral malformation" panel v10.1 and "Retinal disorders" panel v4.0 ((6); (7))

Supplementary References

1. Wiel L, Baakman C, Gilissen D, Veltman JA, Vriend G, Gilissen C. MetaDome: Pathogenicity analysis of genetic variants through aggregation of homologous human protein domains. Human Mutation. 2019 May 22;humu.23798. doi:10.1002/humu.23798

2. Cheng J, Novati G, Pan J, Bycroft C, Žemgulytė A, Applebaum T, et al. Accurate proteome-wide missense variant effect prediction with AlphaMissense. Science. 2023 Sep 22;381(6664):eadg7492. doi:10.1126/science.adg7492

3. Rodrigues CHM, Pires DEV, Ascher DB. DYNAMUT2 : Assessing changes in stability and flexibility upon single and multiple point missense mutations. Protein Science. 2021 Jan;30(1):60–9. doi:10.1002/pro.3942

4. Weisschuh N, Mazzola P, Zuleger T, Schaeferhoff K, Kühlewein L, Kortüm F, et al. Diagnostic genome sequencing improves diagnostic yield: a prospective single-centre study in 1000 patients with inherited eye diseases. J Med Genet. 2024 Jan 19;61(2):186–95. doi:10.1136/jmg-2023-109470.

5. Di Iorio V, Karali M, Brunetti-Pierri R, Filippelli M, Di Fruscio G, Pizzo M, et al. Clinical and Genetic Evaluation of a Cohort of Pediatric Patients with Severe Inherited Retinal Dystrophies. Genes. 2017 Oct 20;8(10):280. doi:10.3390/genes8100280

6. Martin AR, Williams E, Foulger RE, Leigh S, Daugherty LC, Niblock O, et al. PanelApp crowdsources expert knowledge to establish consensus diagnostic gene panels. Nat Genet. 2019 Nov;51(11):1560–5. doi:10.1038/s41588-019-0528-2

7. Caulfield M, Davies J, Dennys M, Elbahy L, Fowler T, Hill S, et al. National Genomic Research Library [Internet]. figshare; 2020 [cited 2026 Feb 26]. p. 5436499 Bytes. Available from: https://figshare.com/articles/dataset/GenomicEnglandProtocol_pdf/4530893/7 doi:10.6084/M9.FIGSHARE.4530893.V7
